# The Organization of the Second Optic Chiasm of the *Drosophila* Optic Lobe

**DOI:** 10.3389/fncir.2019.00065

**Published:** 2019-10-11

**Authors:** Kazunori Shinomiya, Jane Anne Horne, Sari McLin, Meagan Wiederman, Aljoscha Nern, Stephen M. Plaza, Ian A. Meinertzhagen

**Affiliations:** ^1^Howard Hughes Medical Institute, Ashburn, VA, United States; ^2^Department of Psychology and Neuroscience, Dalhousie University, Halifax, NS, Canada

**Keywords:** *Drosophila melanogaster*, optic lobe, optic chiasm, scanning electron microscopy, visual system, medulla, lobula, lobula plate

## Abstract

Visual pathways from the compound eye of an insect relay to four neuropils, successively the lamina, medulla, lobula, and lobula plate in the underlying optic lobe. Among these neuropils, the medulla, lobula, and lobula plate are interconnected by the complex second optic chiasm, through which the anteroposterior axis undergoes an inversion between the medulla and lobula. Given their complex structure, the projection patterns through the second optic chiasm have so far lacked critical analysis. By densely reconstructing axon trajectories using a volumetric scanning electron microscopy (SEM) technique, we reveal the three-dimensional structure of the second optic chiasm of *Drosophila melanogaster*, which comprises interleaving bundles and sheets of axons insulated from each other by glial sheaths. These axon bundles invert their horizontal sequence in passing between the medulla and lobula. Axons connecting the medulla and lobula plate are also bundled together with them but do not decussate the sequence of their horizontal positions. They interleave with sheets of projection neuron axons between the lobula and lobula plate, which also lack decussations. We estimate that approximately 19,500 cells per hemisphere, about two thirds of the optic lobe neurons, contribute to the second chiasm, most being Tm cells, with an estimated additional 2,780 T4 and T5 cells each. The chiasm mostly comprises axons and cell body fibers, but also a few synaptic elements. Based on our anatomical findings, we propose that a chiasmal structure between the neuropils is potentially advantageous for processing complex visual information in parallel. The EM reconstruction shows not only the structure of the chiasm in the adult brain, the previously unreported main topic of our study, but also suggest that the projection patterns of the neurons comprising the chiasm may be determined by the proliferation centers from which the neurons develop. Such a complex wiring pattern could, we suggest, only have arisen in several evolutionary steps.

## Introduction

**FIGURE 1:**
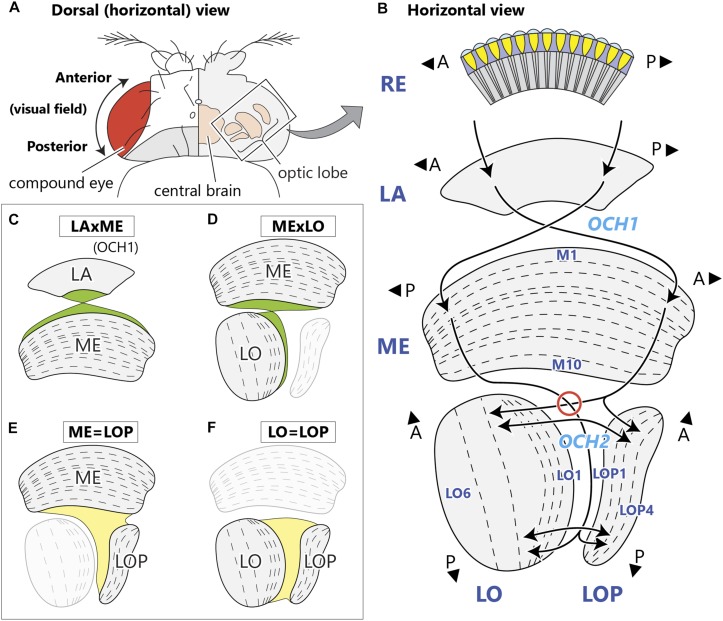
Structure of the optic neuropils and their chiasmata. **(A)** Dorsal view of a fly’s head. The optic lobe forms a large part of the fly’s brain, and is situated lateral to the central brain in the head capsule, just beneath the retina of the compound eye. **(B)** A horizontal schematic view of the optic lobe and the topological projection patterns between the neuropils. The optic lobe comprises four stratified neuropils; the lamina (LA), medulla (ME), lobula (LO), and lobula plate (LOP). The outermost retina (RE) houses ommatidial clusters of photoreceptors that project directly to the lamina. Between the lamina and the medulla, the antero-posterior (A-P) retinotopic axis inverts via the first optic chiasm, OCH1, with the axons of lamina neurons decussating in the middle of the chiasm. Neurons corresponding to the posterior part of the visual field innervate the anterior part of the medulla. From there, neurons relaying the information project to the deepest (proximal) part of the lobula and lobula plate, traveling the longest path in the second optic chiasm, OCH2. Here, axons connecting the medulla and lobula decussate in the chiasm (red circle), while those between the medulla and lobula plate do not. Axons connecting the lobula and lobula plate run in parallel in the chiasm. A and P indicate anterior and posterior, respectively, both corresponding to the retinotopic orientation of the visual field. Names of representative layers according to Fischbach and Dittrich (1989) are shown. **(C–F)** Schematic views of chiasmal fiber projection patterns between the neuropils depicting the projection of OCH1 fibers **(C)**, and corresponding to the three projection patterns of OCH2 neurons **(D–F)**.

Visual input from photoreceptors in the fly’s compound eye is relayed to four neuropils (Figure 1A), the lamina and then medulla, between which the visual field undergoes an anteroposterior inversion, and then two deeper neuropils, the lobula and lobula plate, which face each other at right angles to the medulla. Visual information is then sent to the central brain, in which visual inputs are further processed and integrated with information from other sensory modalities. Each optic lobe neuropil is an array of modular columns, which correspond numerically to the number of ommatidia in the retina. In *Drosophila*, each column of the medulla contains a fixed group of 27 columnar neurons that includes 17 medulla cells and 10 input terminals from the lamina (Shinomiya et al., 2015; Takemura et al., 2015), the latter projecting to the medulla through the first (or outer) optic chiasm (OCH1; Figure 1B; Meinertzhagen and O’Neil, 1991). Axons of the lamina neurons invert their antero-posterior positions by crossing in the first chiasm, resulting in information from the anterior visual field projecting to the posterior side of the medulla, and vice versa. The fibers interconnecting the medulla and lobula complex (LOX) form a more complex structure, the second (or inner) optic chiasm (OCH2; Figure 1B; Meinertzhagen, 1973; Strausfeld, 1976; Ito et al., 2014). Fibers comprising the second chiasm pass in interleaving sheets and bundles of axons, in complex trajectories that have so far eluded detailed reports. Axon sheets pass between lobula and lobula plate, while axon bundles connect the proximal medulla with the distal lobula, inverting the retinal topology in an antero-posterior plane. As a result, the deepest columns of the lobula and lobula plate receive signals from the posterior eye field, in consequence of the two topological crossings in the consecutive chiasmata.

Until now details of the organization of OCH2 have been reported in only a couple of studies. In particular, Figure 9 in Braitenberg (1970) illustrates an extremely high level of geometrical regularity amongst the sheets and interleaving bundles of chiasmal fibers, and the directions of the twist with which these sheets invert their horizontal sequence between medulla and lobula. In total four such sheets and bundles are depicted, each with its own trajectory and twist. These details were derived from light microscopy of silver-stained preparations from larger fly species, presumed to be the housefly *Musca domestica*. Using confocal microscopy, Ngo et al. (2017) visualized the developmental events that give rise to optic lobe neurons, including those constituting the two optic chiasmata, first (OCH1) and second (OCH2), and schematized the projections between the neuropils. They also reported that the neurites that originate the scaffolds of the medulla and LOX are spatially segregated in the OCH2, forming alternating bundled structures surrounded by glial processes.

Here we report more closely and comprehensively details of the structure of the second optic chiasm in *Drosophila*, by analyzing neurons densely reconstructed in three dimensions using focused ion beam-milled scanning electron microscopy (FIB-SEM). With comprehensive knowledge of the neuron classes identified so far, we can provide information on the cell types contributing to each of the axon pathways traversing the chiasm. We also propose in the Discussion some of the potential functional, developmental, and evolutionary consequences of the OCH2 architecture we report anatomically.

## Materials and Methods

### EM Sample Preparation and Imaging

For this study, we used the same optic lobe FIB-SEM data as that reported in Shinomiya et al. (2019). The grayscale data of the image volume are available at http://emdata.janelia.org/optic-lobe/.

### LM Sample Preparation and Imaging

**FIGURE 2:**
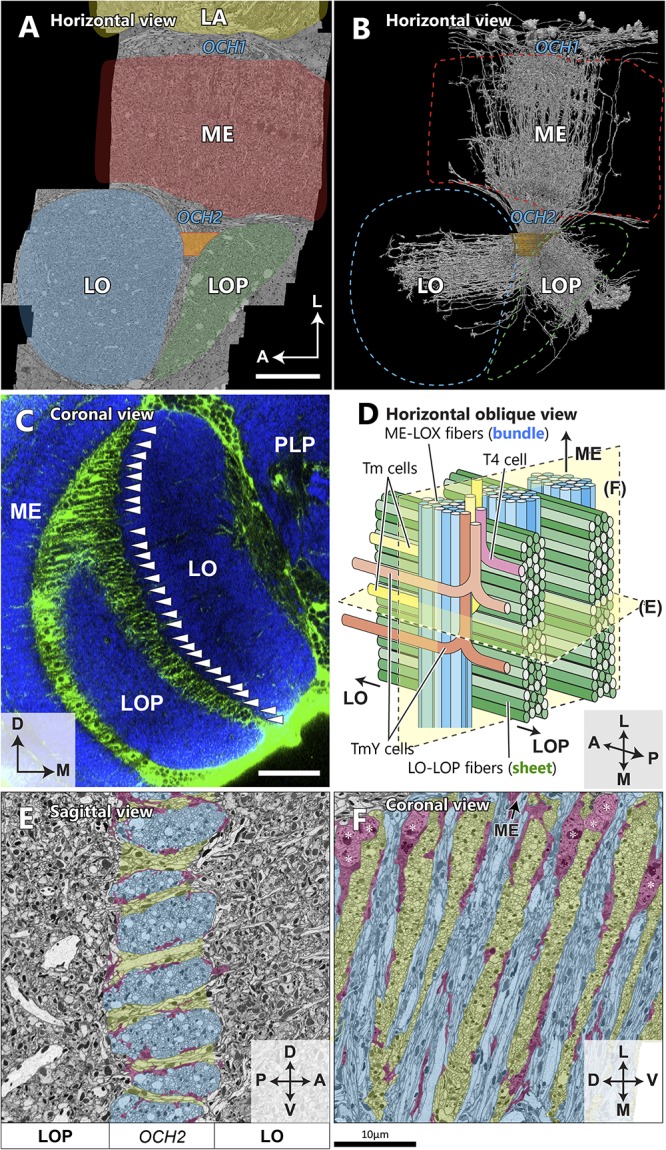
Spatial orientation of the second optic chiasm. **(A)** A re-sliced cross section of the optic lobe imaged with FIB-SEM. The OCH2 region of interest (ROI) used for dense neuron annotation in this study is highlighted in orange. The ROI was set so that it covered 4 bundles and part of 5 sheets, and had a thickness along the L-M axis of 10.5 μm, and a volume of ∼3,870 μm^3^. **(B)** Skeletonized image of 1,225 columnar neurons (∼50% of all those we traced) which intersect the OCH2 ROI. Neurons were randomly selected for visualization. **(C)** A coronal section depicting glial processes in OCH2. Glial tissues (green) are labeled with repo-GAL4>GFP, while synaptic neuropils (blue) are labeled with anti-Brp (nc82 antibody). Arrowheads indicate the bundles of fibers surrounded by glial processes. PLP: posterior lateral protocerebrum. **(D)** A model of the bundles and sheets of OCH2. Fibers in the bundles (shown in blue), running vertically in the panel, connect the medulla and lobula complex, and those in the sheets (shown in green) connect the lobula and lobula plate. Note that axons between the medulla and lobula complex defasciculate from their bundles and merge into the sheets, before projecting to the lobula complex neuropils. The bundle therefore becomes thinner as it runs deeper into the OCH2. Examples of T4 (pink), Tm (yellow), and TmY (orange, brown) cells are in different colors. **(E)** Cross section image of the bundles and sheets, cut perpendicular to the bundles and in parallel to the sheets. The bundles are labeled in blue, the sheets in green, and major glial processes surrounding the structures in red for **(E)** and **(F)**. **(F)** Cross section of the chiasm, cut in parallel to the bundles and perpendicular to the sheets in a plane rotated 90° from that in **(E)**. Cell bodies of glia are indicated by asterisks. Scale bar: **(A,C)** 30 μm, **(E,F)** 10 μm. Axes shown in **(A)**, **(C)**, **(E)**, and **(F)** are based on the body axis.

**FIGURE 3:**
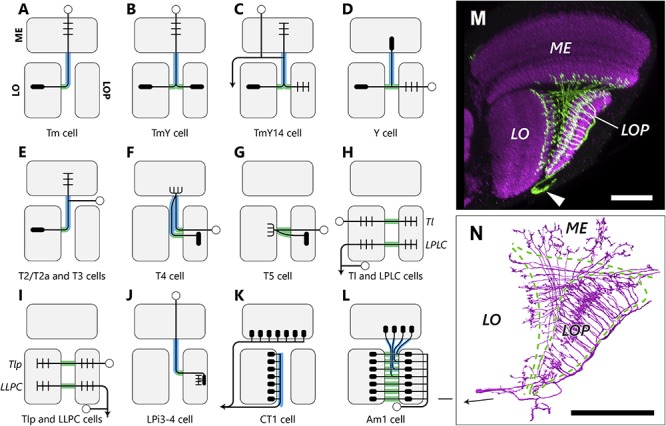
Types and projection patterns of OCH2 neurons. **(A–L)** Neuron types that contribute to OCH2, and their projection patterns. Open circles indicate cell bodies, arrows indicate projections to/from the central brain in **(H)**, **(I)** and **(K)**. Short lines indicate typical input (dendritic/postsynaptic) neurites, and filled ellipses indicate typical output (axonal/presynaptic) neurites. Neuron arbors shown are considerably simplified, and pre- and postsynaptic motifs almost always coexist at single neurites in actual neurons, so that input and output sites are therefore not strictly segregated. Lines shown in green and blue, respectively, correspond to neuronal fibers in the bundles and sheets. **(M)** The Am1 cell visualized with FLP-out. Arrowhead indicates the cell body. **(N)** To compare with **(M)**, Am1 reconstructed from the FIB-SEM dataset. The arrow in **(N)** indicates the cell body fiber. Scale bar: **(M,N)** 30 μm.

To visualize glial processes surrounding the chiasm bundles (Figure 2C), we used genetic means to combine a pan-glial repo-Gal4 driver (Lai and Lee, 2006; Awasaki et al., 2008) with a membrane bound UAS-mCD8-GFP reporter (FlyBase ID: FBtp0002652) (Lee and Luo, 1999). As a background label we also used immunolabeling with anti-Bruchpilot (Brp), utilizing mAb nc82 (RRID: AB_2314866) (Wagh et al., 2006; Hofbauer et al., 2009) from the Developmental Studies Hybridoma Bank. Samples were imaged as previously described (Otsuna and Ito, 2006; Ito et al., 2014), and the brightness adjusted using an image-processing program, Fiji (Schindelin et al., 2012). The Am1 cell (Figure 3M) was visualized using MultiColor FlpOut (MCFO), by crossing a driver line VT048141 to MCFO-2, and imaged as described previously (Nern et al., 2015; Shinomiya et al., 2019).

### Reconstruction and Annotation of Neurons

Neurons in the FIB-SEM dataset were reconstructed using a tracing and visualization tool, NeuTu^1^ (Feng et al., 2015). Neurons were identified and classified by their morphology according to previous neuroanatomical reports of optic lobe neurons (Fischbach and Dittrich, 1989; Hausen and Egelhaaf, 1989; Shinomiya et al., 2015; Panser et al., 2016; Klapoetke et al., 2017).

## Results

### Repetitive Structures of the Second Optic Chiasm

The FIB-SEM image volume we used for this study covers the entire depth of the medulla, lobula, and lobula plate, as well as the proximal part of the lamina (Figure 2A; Shinomiya et al., 2019). We set a region of interest (ROI) covering part of the second optic chiasm (Figures 2A,B), and traced all neurons passing through the ROI to uncover the structure of the second optic chiasm. OCH2 comprises two repetitive components: bundles and sheets (Figures 2C–F). Neurites interconnecting the medulla and the lobula complex are tightly packed all together into bundles. Each bundle is surrounded by glial processes (Figures 2C,E,F), and separated from its neighbors as well as from surrounding neurites (Ngo et al., 2017). Most cell bodies of glia in the chiasm are located in the area close to the medulla (Figure 2F), while their processes are distributed throughout the entire thickness of the chiasm. The glial processes surrounding the bundles are not densely distributed, and as a result the bundles are not completely wrapped by glia (Figures 2E,F). The bundles contain axons of various types of neurons as well as cell body fibers (of T4 cells, for example). Columnar neurons such as transmedullary (Tm) and TmY cells, for example, that are from the same medulla column, project to the same column in the lobula as well, but interestingly do not necessarily run through the same bundle, thus desegregating in the chiasm. One bundle contains neurons from multiple columns. Typically, there are about 25–30 bundles in an optic lobe, counted using repo-GAL4>GFP staining and confocal scanning light microscopy. The thickness of a bundle, or the number of axons within a bundle, is not constant, and one bundle may be twice as thick as another. A single bundle is thickest when it exits the medulla, and thins as it runs deeper into the chiasm and as axons leave the bundle and project to their corresponding positions in the lobula and lobula plate (Figure 2D).

Connecting the lobula and the lobula plate, neurites, which include both axons and cell body fibers, form sheet-like structures. These sheets are also surrounded by neuropil glia and physically separated from the bundles (Ngo et al., 2017). Most of the cell bodies of the glia are located in the chiasm at the proximal surface of the medulla, though some cell bodies are also found in the chiasm deeper, on the lobula complex side. Neurites running through the bundles (e.g., those from T4, Tm, and TmY cells) merge into the sheets before projecting to the lobula or lobula plate (Figure 2D). The rows of bundles, together with the sheets in between, physically separate the lobula from the lobula plate. In a cross-sectioned view, a bundle appears to be an oval shape flattened in the dorsoventral axis (Figure 2E). The sheets, on the other hand, occupy the space between the bundles and are continuous seamlessly for the entire lateral to medial thickness of the lobula complex (Figures 2D,F). Anatomically these two types of structure of the chiasmal fibers are thus distinct from each other.

### Component Neuronal Fibers of the Chiasm

While without exception all axons passing through OCH1 between the lamina and medulla cross and invert their relative positions about the antero-posterior axis (LAxME type; Figure 1C), neurons traversing the OCH2 pursue more complex trajectories that can be further categorized into several groups according to their projection patterns. Axons connecting the medulla and lobula decussate within OCH2 (MExLO type; Figure 1D), inverting the antero-posterior axis of the visual field. Other groups include neurons connecting the medulla and lobula (ME=LOP type; Figure 1E), and the lobula and lobula plate (LO=LOP type; Figure 1F). These neurons connect the neuropils with axons that all have parallel trajectories, conserving the axes of the visual field before and after the projection. There are also neurons innervating all three neuropils, which have axons that branch within OCH2 and project to corresponding columns in each neuropil, conserving retinotopy in all of these neuropils. The projection pattern of these neurons can be considered as a combination of MExLO and ME=LOP types.

By tracing all neurons intersecting with the ROI (Figures 2A,B), we have annotated 2,563 neurons in total in the ROI (Table 1). The estimated numbers of cells in the entire optic lobe were also calculated based on the coverage rate of the ROI. Most neuron types constituting the chiasm are columnar neurons connecting the several neuropils of the optic lobe, while there are a few tangential cells that have much broader arbors in each neuropil than those of the columnar cells. We have identified the following neuron types in OCH2, and the innervation patterns of these different types of neurons in the three neuropils are shown in Figures 3A–L.

**TABLE 1:** Cell counts by neuron type in OCH2 ROI and an estimate for the entire optic lobe.

**Neuron type**	**Projection type**		**Cell count**	**Total**
			**in ROI**	**estimate**
Tm	MExLO	Figure 3A	1, 276	9, 251
T4	ME=LOP	Figure 3F	397	2, 878
TmY	MExLO+ME=LOP	Figure 3B	396	2, 871
T5	LO=LOP	Figure 3G	165	2, 878
Y	ME=LOP+LO=LOP	Figure 3D	66	479
T3	MExLO	Figure 3E	56	406
T2/T2a	MExLO	Figure 3E	47	341
LLPC/Tlp	LO=LOP	Figure 3I	37	645
TmY14	MExLO+ME=LOP	Figure 3C	25	181
LPLC/Tl	LO=LOP	Figure 3H	13	227
DCV	*Various*	*not shown*	9	
LPi3-4	ME=LOP	Figure 3J	5	36
CT1	ME, LO	Figure 3K	1	1
Am1	ME=LOP+LO=LOP	Figure 3L	1	1
Orphan or other			69	
Total			2, 563	20,195

*Neurons were densely traced and annotated within a region of interest (ROI) covering part of OCH2 (see Figures 2A,B); numbers of identified cells within the ROI were then counted. “DCV” includes neurons with dense core vesicles in the terminals, and these had various different projection patterns; “orphan or other” includes bodies that could not be fully traced or whose neuron types could not be identified. Corresponding panels in Figure 3 are indicated for each cell type. See the main text for details.*

#### Medulla – Lobula Complex Neurons

##### Tm and TmY cells

Tm and TmY cells extend axons from cell bodies in the medulla cortex (Figures 3A,B; Fischbach and Dittrich, 1989). These penetrate the medulla, usually giving rise to a columnar arbor in specific strata M1-M10, then project through the chiasm to the lobula complex (Tm cells to the lobula and TmY cells to the lobula and lobula plate). Their axons form bundles roughly ensheathed by glial cell processes and separated from surrounding fibers and synaptic terminals. Axons of Tm cells defasciculate from the bundles and project to the lobula, whereas axons of TmY cells bifurcate within the chiasm and their branches project to both neuropils of the lobula complex. Within a single bundle, spatial topology, both the anteroposterior or dorsoventral organization, in the medulla is mostly conserved. For example, fibers from anterior medulla columns occupy the anterior part of the bundle, and those from posterior columns pass to the posterior part (Figure 2D). Fibers run mostly in parallel in a bundle, and Tm cell axons decussate when they exit from the bundle, just before projecting to the lobula. After leaving a bundle, fibers pass between the bundles, merging into sheet-like structures composed of other lobula-lobula plate neurons. Axons of most Tm and TmY cells look alike in the chiasm and are about 100–300 nm in diameter. We find large and small axon profiles in cross-sectioned chiasmal bundles (Figure 2E) but no clear correlate of the regular alternation of large with small caliber fibers reported by Braitenberg (1970) for, presumably, *Musca*.

The cell TmY14 (Takemura et al., 2013) is actually a visual projection neuron (VPN) morphologically distinct from other TmY cell types (Figure 3C). While the cell body resides in the medulla cortex, its cell body fiber penetrates the medulla without branching or making synapses, and bifurcates at the surface of the proximal medulla. One of the neurites further branches into multiple processes, which project to the medulla, lobula, and lobula plate neuropils. The other neurite projects to the central brain (A. Nern, unpublished observation). TmY14 cells have notably thicker axons (about 700–1500 nm in diameter) that are easily distinguishable from other fibers in the chiasm, and may correspond to the large-caliber axon profiles depicted by Braitenberg (1970). Unlike other TmY cells, each TmY14 cell may contribute up to three axons in the chiasm, at least one of which projects to both the lobula and lobula plate.

##### Y cells

Cell bodies of the Y cells lie in the lobula plate cortex. They project to all of the lobula plate, lobula, and proximal medulla (Figure 3D). Y cells largely share the same trajectory as TmY cells. Although pre- and postsynaptic sites are more or less mixed at every location, most input sites onto Y cells are in the lobula plate, while those of TmY cells are in the medulla. Axons of different Y cell types seem to be distributed randomly in the chiasmal bundles and are not clustered. Typical Y cells, such as those illustrated by Fischbach and Dittrich (1989), have only one axon in the chiasm and project to the medulla, while we also encountered some types of Y cells with multiple axons in the chiasm, between the lobula complex and the medulla. Most of these cells seem to be novel Y cell subtypes. Multiple axons of a single neuron may be distributed in a single bundle, or physically separated into multiple bundles.

##### T2/T2a and T3 cells

The T2/T2a and T3 cells all have their cell bodies between the medulla and lobula, posterior to these neuropils (Figure 3E; Fischbach and Dittrich, 1989). During development, these cells, along with C2 and C3 cells, derive from a larval cell population called the distal cell group, which arises from the inner proliferation center (IPC) of the optic lobe (Hofbauer and Campos-Ortega, 1990; Apitz and Salecker, 2015, 2018). The cell body fiber travels along the proximal surface of the medulla, bifurcates in this area, and one branch then projects to the medulla, while the other projects to the lobula, as part of a bundle, with an inversion of the A-P axis. T2 and T2a extend their medulla branches up to layer M1, while T3 terminates within the proximal medulla. Since T2 and T2a neurons are morphologically similar except differing in their neuronal connections, these two subtypes are not distinguished in this study.

##### T4 cells

T4 and T5 cells are directionally selective neurons that have been intensively studied as part of the fly’s motion detection circuits. The numerous cell bodies of T4 cells are located in the lobula plate cortex (Figure 3F). A T4 cell body fiber penetrates all strata of the lobula plate without making synapses, and projects through a bundle to the most proximal, tenth layer of the medulla (M10), where it forms synaptic contacts with the medulla intrinsic (Mi) and Tm cells. It then makes a 180° turn to project back to a lobula plate layer, again through a chiasmal bundle. The cell body fiber and the axon projecting from the medulla to the lobula plate thus both traverse the chiasma, but are spatially separated in the bundles, often passing like strangers in different bundles, to and from the medulla. T4 cells are further categorized into four subtypes, T4a, T4b, T4c, and T4d, depending on the target layer to which each projects in the lobula plate (Takemura et al., 2017). Each subtype is closely matched to the others, having equal numbers and synaptic inputs to those in the other three, and each projects to its own layer, and is sensitive to a different direction of stimulus motion. T4 dendrites receive inputs from upstream Mi and Tm cells in M10, and these much-studied cells have been reported to function as the elementary motion detector (EMD) in the ON-edge motion detection pathway (Joesch et al., 2010; Takemura et al., 2013, 2017; Borst, 2014; Shinomiya et al., 2019).

#### Lobula – Lobula Plate Neurons

##### T5 cells

T5 cells, like the T4 cells, which they complement, have cell bodies in the lobula plate cortex (Figure 3G). Their cell body fibers penetrate the lobula plate, pass in the sheets of the chiasm and form synaptic contacts at the first layer of the lobula (Lo1). There they make a turn to project to a specific lobula plate layer. The cell body fiber and its axon may or may not be physically adjacent in the sheets. The T5 cells receive inputs in the most proximal lobula layer, Lo1, and function as the EMD in the OFF-edge motion pathway (Joesch et al., 2010; Borst, 2014; Shinomiya et al., 2014, 2019).

##### LPLC and Tl cells

The lobula plate – lobula columnar (LPLC) cells are VPNs projecting from the lobula complex to the central brain (Panser et al., 2016; Klapoetke et al., 2017). They have cell bodies in the lateral cell body rind (LCBR) between the optic lobe and the central brain neuropils. Their primary neurites branch into two, one projecting to the lobula passes through a sheet of the chiasm and terminates in the lobula plate. The other branch projects to a neuropil in the central brain (Figure 3H). The projection of translobula (Tl) cells (Fischbach and Dittrich, 1989) is similar to LPLC cells within the optic lobe, but these cells lack the central brain projection. Tl cells are lobula neurons, with cell bodies in the lobula cortex, adjacent to the proximal surface of the lobula. LPLC cells and Tl cells were not always distinguishable in our study, because the image data covers only the optic lobe.

##### LLPC and Tlp cells

The projection pattern and morphology of the lobula – lobula plate columnar (LLPC) cells and translobula-plate (Tlp) cells (Fischbach and Dittrich, 1989) are similar to the LPLC/Tl cells but in a retrograde direction, insofar as they project to the lobula plate first and terminate in the lobula (Figure 3I). LLPC cells are VPNs with their cell bodies in the LCBR, and Tlp cells are lobula complex interneurons with cell bodies in the lobula plate cortex.

#### Other Cell Types

##### LPi3-4 cell

The lobula plate houses many types of lobula plate intrinsic (LPi) cells. Most of these have their cell bodies in the lobula plate cortex, and only one subtype, the LPi3-4 cell, has its cell body in the medulla cortex and projects to the lobula plate through OCH2 (Figure 3J). LPi3-4 is a cell type connecting the layers Lop3 and Lop4 of the lobula plate, and is associated with the processing of vertical motion information (Mauss et al., 2015). It has input from (is postsynaptic to) sites specifically in Lop3 and output (presynaptic to) sites in Lop4 that transfer information unidirectionally from its upstream T4 and T5 cell terminals in Lop3 to the downstream vertical system (VS) cells in Lop4 (Mauss et al., 2015; Shinomiya et al., unpublished observation). Each LPi3-4 cell receives inputs from multiple T4 and T5 cell terminals, covering an area equivalent to a few columns in the lobula plate. Even though its cell body fiber penetrates the medulla and OCH2, no synapses are found outside the two target layers of the lobula plate.

##### Tangential/amacrine cells

We have found three large tangential elements that pass through OCH2 and innervate multiple neuropils in the optic lobe.

**FIGURE 4:**
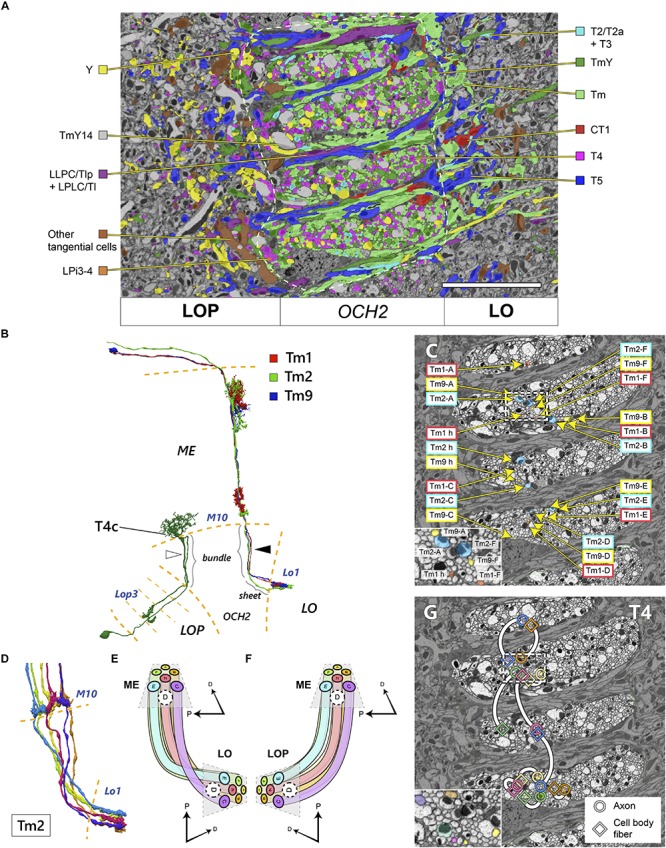
Topology of neurons in OCH2 pathways. **(A)** Distribution of different cell types in a cross-section of OCH2. “Am1 + tangential cells” include Am1 and other tangential elements including those not contributing to the chiasm. Neurons with dense core vesicles (DCV) are not color-coded. See also Table 1 and Figure 2E. White dashed line demarcates the area of OCH2 in which neurons are densely traced and annotated. **(B)** Reconstructed Tm cells and a T4c cell. Three Tm cells, Tm1, Tm2, and Tm9, innervate the same columns both in the medulla (ME) and lobula (LO), but are not tightly bundled in the chiasm (arrowhead), see text. The T4 cell has two fibers in the chiasm, a cell body fiber and an axon connecting the medulla and lobula plate (LOP; in this case the third layer, Lop3). Fibers contributing to the bundle or sheet in the chiasm are so indicated. These fibers do not necessarily run next to each other in the chiasm either (white arrowhead). **(C)** Axons of the Tm1, Tm2, and Tm9 cells from seven columns in OCH2. The neurons are color-coded by neuron types. The inset shows an enlarged view of part of the chiasm, indicated by a dotted box. **(D)** Six reconstructed Tm2 cells, a typical one-per-column neuron, in the chiasm from M10 to the lobula terminals. **(E,F)** Projection patterns of columnar neurons connecting the medulla and lobula **(E)** and the medulla and lobula plate **(F)**. The spatial layout of the neurons as well as the color code of **(E)** are the same as for the panel **(D)**. **(G)** Distribution of the axons and cell body fibers of T4 cells in OCH2. Circles indicate the axons, and squares indicate the cell body fibers. Each pair of profiles having the same color and connected with a white line belongs to a single T4 cell. The inset shows an enlarged view of part the chiasm, indicated by the dotted rectangle. Scale bar: **(A,C,G)** 10 μm. D, dorsal, P, posterior.

(1) The CT (complex tangential) cell, CT1, is a unique tangential neuron that primarily innervates the M10 and Lo1 layers of the medulla and lobula, respectively (Shinomiya et al., 2015, 2019; Takemura et al., 2017). A pair of cell bodies is located in the central brain, and each cell covers the entire M10 and Lo1 strata of the optic lobe. The axon enters the optic lobe from the posterior side, at a point close to the entry site to the optic lobe of the posterior optic commissure fibers, and branches into the medulla and lobula pathways. The medulla pathway passes between the lobula and the central brain and projects to the M10 layer, whereas the lobula pathway projects to the Lo1 layer via the bundles of the chiasm. The axons projecting to the Lo1 layer run next to the lobula neuropil within the bundles (Figure 3K; also see Figure 4A). The lobula pathway further branches into multiple axons, and projects to each column of the Lo1 layer (Figure 3K). Other than the columnar terminals innervating the lobula, a few tangential branches project to the lobula and lobula plate, and also through the chiasm. CT1 is assumed to be involved in the motion information processing circuit along with T4 and T5 in the M10 and Lo1 layers, respectively, where they form columnar terminals that contribute to the compartmentalization of neuronal computation of motion information (Meier and Borst, 2019; Shinomiya et al., 2019).

(2) Am1 is an amacrine-type local neuron that innervates all three neuropils (Figures 3L–N). The cell was initially reported by Hausen and Egelhaaf (1989) in the optic lobe of *Calliphora*, and has a cell body in the posterior part of the optic lobe, next to the lobula plate (Figure 3M). It enters the lobula plate from its posterior side and branches into columnar fibers and forms synaptic sites mainly in the Lop2 layer. The fibers then bifurcate and project through OCH2 to M8 and M9 of the medulla and to Lo2 of the lobula. Fibers connecting the lobula plate and medulla pass through the bundles, and the others project to the lobula via the sheets (Figures 3M,N). Interestingly, only the posterior part of the medulla, which receives information from the anterior, frontal part of the visual field, is innervated by this cell (Figure 3M). It is also notable that a few Am1 fibers within the bundle terminate within the chiasm, making synapses with other neurons (see section “Ultrastructure in the chiasm”).

(3) We also have found another tangential element that innervates the three neuropils. We did not give it a specific name since the cell’s branches were insufficiently traced to enable us to identify this cell uniquely, but its reconstructed terminals were predominantly presynaptic, suggesting the cell is a centrifugal VPN.

##### Pure chiasmal neurons (neurons terminating in the chiasm)

We also found a few (3 in 2,563) annotated neurons that originate in the medulla and terminate within the chiasm, without projecting to a target neuropil. In some cases, they form synaptic contacts with surrounding neurons around their point of termination, although it is not common for neurons to form synapses in the chiasm. We have insufficient data, though, to confirm whether these are stable, truly functional neurons, or possibly growing or retracting neurites, albeit that may be in arrested growth.

Given that the region of interest (ROI) we used to count numbers of neurons covered only part of the chiasm, we tried to estimate the total numbers of the neurons in the whole optic lobe by means of extrapolation. The entire OCH2 has ∼28 bundles on average (*n* = 3). Since the ROI covers four bundles, the total numbers of neurons passing through the bundles are estimated by multiplying the counted numbers by 28/4 (e.g., T4 cell: 397 cells x 28/4 = 2,779 cells). The coverage rate for the bundles is 4/28 × 100 ≈ 14.3%. We then assumed that the numbers of T4 and T5 cells in the optic lobe are the same, and calculated the coverage rate for the sheets as 165/2,779 × 100 ≈ 5.9%. The numbers of neurons that only contribute to the sheets (neuron types with asterisks in Table 1) are estimated from the coverage rate. We have found a handful of cells and neurite fragments with dense core vesicles (DCV); these have various different projection patterns and therefore we did not estimate their total number. In total, for the others we predicted that there are approximately 19,500 cells per hemisphere that contribute to OCH2, and that of these the Tm cells contribute the most. Since there are an estimated 100,000 neurons in the fly’s brain (Shimada et al., 2005; Scheffer and Meinertzhagen, 2019), and about 30,000 neurons per hemisphere among them are optic lobe neurons (Meinertzhagen and Sorra, 2001; Shimada et al., 2005; Ito et al., 2013; Zheng et al., 2018; personal communication: K. Ito and T. Shimada), about two thirds of the optic lobe neurons are likely to contribute to the second optic chiasm. We presumed that there are 2,779 cells for each of T4 and T5, a number consistent with the report that the total number of T4 and T5 cells is about 5,300 per hemisphere (Mauss et al., 2014). Since we reconstructed and annotated neurons only within the ROI, there might be unidentified types of neurons outside the ROI that are not listed in Table 1. Examples of cell types that are excluded are neurons specifically innervating dorsal/ventral regions of the neuropils. Besides such exceptional cases, however, we think we have identified all typical cell types that project to the main part of the neuropils we have analyzed.

### Topology of Chiasmal Pathways

We mapped neurites within the chiasm according to their neuron types, to examine the chiasmal topology proposed by Braitenberg (1970). In Figure 4A, neurons in the OCH2 ROI are color-coded by their cell types. Axons of Tm, TmY, and T4 cells (including the cell body fibers) are of a similar caliber, approximately 200–400 nm in diameter. Axons of Y cells and TmY14 cells are significantly thicker than these, about 700–1,200 nm in diameter. Together with all other neuronal fibers going through the bundles, the fibers are intermingled and almost evenly distributed in caliber, without obvious clustering of fibers having a similar caliber. Our findings do not support those proposed by Braitenberg (1970) for *Musca*. In the sheets between the bundles run the fibers connecting the lobula and lobula plate, but fibers that exit the bundles and project to the lobula complex neuropils also merge into the sheets. While glial processes loosely surround the bundles and separate fibers in the bundle from those in the sheets, neurites exiting the bundle make a 90° turn and pass the glial limit to merge into the sheet. The fibers of these neurons, including the Tm, TmY, Y, and T4 cells, always leave the bundles on the side close to the lobula plate before projecting to the lobula complex. Axons from the bundle projecting to the lobula, i.e., Tm, TmY, Y, T2/T2a, and T3, therefore run the entire thickness of the sheets from the lobula plate side to the lobula side, while those projecting only to the lobula plate, i.e., T4 and LPi3, project almost directly to that neuropil, contributing little to the sheets. The branching of neurites of neurons projecting to both the lobula and lobula plate, including TmY and Y cells, almost always occurs in the sheet after the axon coming from the medulla exits the bundle. During the larval and early pupal stages, axons of the T2 and T3 cells are bundled altogether and are segregated from the axons of the Tm cells (Ngo et al., 2017), whereas axons of these cells were intermingled in the adult and no spatial segregation was observed in the chiasm (Figure 4A). Some rearrangement is therefore implied by this difference.

Fibers of the tangential cells run unique trajectories in the chiasm. For example, fibers of CT1, which lead to the columnar terminals in the lobula, always run proximally to the lobula in the bundles. The axon of CT1 branches in the bundles, and it often merges into a sheet and makes a 180° turn there, before projecting to a target column in the lobula, making a characteristic “loop” of the axon (Shinomiya et al., 2015, 2019; Takemura et al., 2017).

Next, we examined the topology of individual axons in OCH2 bundles. Three types of Tm cells, Tm1, Tm2, and Tm9, are medulla columnar cells which project to lobula layer 1 (Lo1) (Figure 4B; Fischbach and Dittrich, 1989). They are known to be presynaptic to T5 cell dendrites in Lo1, constituting part of the EMD of the OFF-edge motion-detecting pathway (Joesch et al., 2010; Shinomiya et al., 2014, 2019). Since each medulla and lobula column houses a single subset of these three Tm cell types, we traced axon triplets to corresponding columns in the medulla and lobula. Axons of the three Tm types in seven columns, h (home), A–F, are shown in Figure 4C. The columns are oriented in a hexagonal array in both neuropils [see Figures 4D–F; also see Takemura et al. (2017) and Shinomiya et al. (2019) for reference]. The axons largely preserve their spatial orientation in the bundles, though axons from the same column are not tightly bundled together, sometimes even running through different bundles (axons of h, A, and C columns in Figure 4C). The columns are connected through the fibers in the bundles as shown in Figures 4D,E (note that, for clarity, the fibers in column D are not shown). The fibers run mostly in parallel in the bundles of OCH2, conserving the topology of the visual field, and make a cross when they exit the chiasm and project to the target columns of the lobula. The hexagonal array, as well as the spatial orientation of the columns, is unchanged before and after the projection. The axons connecting the medulla and lobula plate (e.g., TmY or T4 cells), in contrast, lack decussation while nevertheless maintaining the spatial orientation of the visual field (Figure 4F).

We further mapped the neuronal fibers of the T4 cells in the bundle. T4 has a cell body fiber and an axon running through the chiasm (Figure 4B). While the cell body fiber does not have synapses, it runs in parallel to the axon connecting the dendritic input sites in medulla layer 10 (M10) with the terminal in the lobula plate. It turns out that in some cases the axon runs very close to the cell body fiber in the same bundle, while in other cases they run apart and even in the different bundles (Figure 4G). Similarly, in the case of the T5 cells, the axon and the cell body fiber can populate either the same sheet or different sheets (data not shown). Moreover, even if dendrites of multiple T4 or T5 cells are located close to each other in M10 or Lo1, their axons or cell body fibers could run several bundles/sheets apart from each other in the chiasm. This finding suggests that the positions of the axon and the cell body fibers of T4 and T5 are not strictly correlated, or that the two fibers are not likely to interact with each other during development, for example by selective fasciculation.

We may summarize these observations as revealing that the trajectories of axons in the chiasma reveal no very strict organization into tight bundles, unlike those same axons have when entering and leaving that tract. On the contrary they appear to meander somewhat within the chiasm, bundling tightly only at either end.

### Ultrastructure in the Chiasm

**FIGURE 5:**
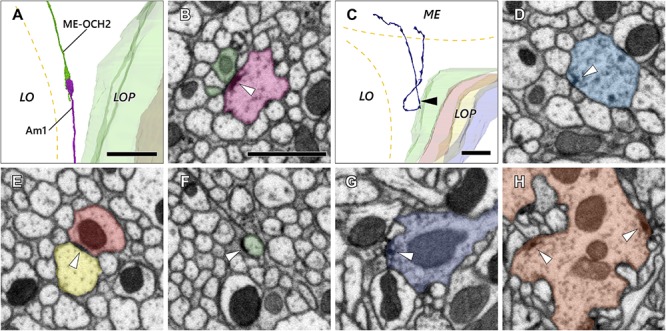
Axon and synaptic ultrastructure in the chiasm. **(A)** An Am1 branch and a ME-OCH2 cell terminal, both terminating in OCH2. **(B)** The two cells shown in **(A)** form a synaptic contact within OCH2. The two sites of synaptic contact are revealed where the Am1 terminal (red), forms a T-bar ribbon (arrowhead), which is presynaptic, opposite the ME-OCH2 terminal (green) where it is postsynaptic. **(C)** Part of a DCV cell making a reverse turn in OCH2. Its ME part is incompletely reconstructed. **(D)** A synaptic site of the DCV cell shown in **(C)**. The synaptic site corresponds to the point indicated by the arrowhead in **(C)**. The DCV cell has a presynaptic T-bar (arrowhead) and provides input to a dyad of postsynaptic Tm cell axons. **(E,F)** Putative septate or adherens junctions in OCH2. **(E)** is a case between Tm cell axons, and **(F)** is a case between a Tm cell axon and glia. **(G,H)** Typical presynaptic T-bars found outside OCH2. **(G)** is an example of a Tm1 cell, while (H) is a Tm9 cell terminal, both in the first stratum of the lobula. Scale bar: **(A,C)**: 10 μm; **(B)**, **(D–H)**: 1 μm.

The second optic chiasm (OCH2) is, in general, a synapse-free structure composed of axons and cell body fibers. There are, however, a few exceptions among cells that form synapses or related junctions between neurons in the chiasm. One notable example is the cell Am1 (Figures 5A,B, also see Figures 3M,N), which extends fibers from the lobula plate to the lobula and the medulla, with a small portion of the fibers terminating within OCH2. There the terminal forms a synaptic contact with a presynaptic organelle (a “T-bar” ribbon) onto a neurite extended from the medulla that terminates in the chiasm (ME-OCH2 cell; Figures 5A,B). The ME-OCH2 cell is not among the known OCH2 cell types, and neurons of this novel cell type are apparently not regularly distributed since only two such cases were found in the examined region of interest. Each ME-OCH2 cell had only one synaptic terminal in the chiasm, and received inputs exclusively from Am1 in each case. Since the examined region of interest covers approximately 14% of the entire bundles (Table 1), if evenly distributed we would predict to find only about 15 of this cell type in the entire chiasm, assuming the cells are evenly distributed.

In another case, a bouton-like terminal filled with larger vesicles (approx. 100–150 nm in diameter) had T-bar ribbons onto other fibers in the chiasm (Figures 5C,D). The appearance of the vesicles was consistent with primary lysosomes (Novikoff et al., 1956; Stark et al., 1988; Hurbain et al., 2017). This neuron originates in the medulla, enters OCH2 and makes a 180° turn in the chiasm before returning to the medulla (Figure 5C). Its further innervation pattern in the medulla or the cell body site could not be identified because part of the cell exited the volume, and no other neurons with a similar morphology have been found in the dataset. This neuron was notable because, unlike other neurons, it actually formed multiple bouton-like structures in the chiasm. The neuron has about 10 such bouton-like terminals along its axon and each terminal has 1–3 output synapses.

Disk-shaped, junction-like structures were also observed in the chiasm (Figures 5E,F). These are 200–500 nm in size and they look distinct from regular synaptic sites with T-bar ribbons (Figures 5G,H) insofar as they lack a presynaptic pedestal and platform. In Figure 5E, the structure was seen between two axons and the density was distributed symmetrically across the membranes. It resembles the septate or adherens junctions (Matter and Balda, 2003) that are found widely among epithelial cells both in insects and vertebrates but also in neuronal tissues (Tepass and Harris, 2007). In Figure 5F, on the other hand, the structure was found between an axon and a glial cell, and the density was asymmetrically distributed on the axon’s side. This type of ultrastructure is occasionally found outside of the chiasm as well, especially in the proximal medulla and distal lobula, in association with motion-sensitive T4 and T5 cell dendrites (data not shown). The structure appears to be similar to a hemidesmosome, which has been reported in wing epidermal cells in *Drosophila* (Mogensen and Tucker, 1987). In vertebrates, integrin α6β4, a protein component of the hemidesmosome, is widely expressed in epithelial cells, and also known to be expressed as part of non-epithelial cells including neurons and glia (Walko et al., 2015).

It is unclear if these ultrastructure features, especially the synaptic motifs, are functionally meaningful, especially because they are distributed very sparsely in the chiasm and apparently outnumbered by synaptic contacts outside the chiasm. These observations, however, may suggest that the chiasm is not merely a bundle of fibers, but also a functional neuropil modulating visual information dynamically at least in a certain period of its development or at a particular stage of life.

## Discussion

### Developmental Background of the Second Optic Chiasm

We report the presence and organization of the two patterns of axon trajectory, in sheets and bundles, respectively, within the second optic chiasm of *Drosophila*, and the cell types that contribute these. We have been unable to confirm the chiasmal organization reported by Braitenberg (1970) for *Musca*. The topology we report immediately poses two major questions concerning their ontogenetic origins: how do these trajectories arise during development and, further, how did they arise in evolution.

First, given that at other sites axons project between neuropil regions only when guided by the trajectories of those that have grown previously, by pioneering and fasciculation (Lin et al., 1994; Hidalgo and Brand, 1997), in what sequence of outgrowth do the axons of all three neuropils innervate their respective columns? The compound eye, which innervates the lamina and medulla, develops as a wave of differentiation that spreads across the ommatidial eye field from its posterior edge (Meinertzhagen, 1973; Meinertzhagen and Hanson, 1993). We, therefore, need to compare axon growth in columns of the three neuropils that correspond chronotopically.

Second, how could such a complex system have arisen during the course of evolution? Any attempt to make sense of the OCH2 in flies has to reconcile any proposed simpler ancestral forms, insofar as these are still represented in extant groups (Strausfeld, 2012). They have yet to be reported clearly or in sufficient detail, however, making close comparisons difficult. Ancestral groups such as dragonflies have been reported with a single prominent lobula neuropil (Zawarzin, 1913), but lack a clear counterpart to the dipteran lobula plate, for example.

Although no direct evidence has been shown so far about the growth order of the fibers comprising the second optic chiasm, some features can be speculated upon from the anatomical structures revealed in this study and from knowledge derived from related developmental studies.

In the lamina and distal medulla, each pair of lamina cartridges or medulla columns together contains a complete subset of the photoreceptors (R1-R6, R7 and R8), lamina cells (L1-L5), T1, C2, and C3 cells, all of which have columnar neurites (Fischbach and Dittrich, 1989; Takemura et al., 2017). In OCH2, however, far fewer classes of neurons have a single representative per column; these do, however, include Tm1, Tm2, Tm9, T2, T2a, and T3 (Takemura et al., 2017; Shinomiya et al., 2019). Among these, only the terminals of the three Tm cell types form clear columnar structures in the lobula that correspond one-to-one with the ommatidia (Shinomiya et al., 2014, 2019). Given their singularity we consider them to be the lobula’s building blocks, and propose that they are first to innervate that neuropil, and thus to pioneer the second chiasm. Other columnar neurons connecting the medulla and lobula complex via the bundles (e.g., other Tm cells, TmY, Y, T4 cells, etc.) do not have a strict one-to-one relationship with the ommatidia, or have fewer cells than the number of the medulla/lamina columns. The latter fact strongly implies that the retinotopy of the lobula and lobula complex may be determined by projections of this first group of Tm cells (Tm1, Tm2, Tm9) from the medulla, one-per-column, and that it is these cells that project to the lobula complex first among the neurons comprising the bundles, and that thus possibly establish the retinotopic map of its elements. The axons of the other cell types, we suggest, grow and fasciculate along these. Their defasciculation in the chiasm, in contrast to their grouping when they enter or leave the neuropils, suggests that the chiasm extends by passive displacement only after axons have grown between fixed points of entry to closely bordering neuropils that then separate.

Optic lobe neurons develop from outer (OPC) and inner proliferation centers (IPC), two distinct areas of the neuroepithelium (Apitz and Salecker, 2015, 2016, 2018; Ngo et al., 2017). The OPC produces two neuroblast populations that differentiate in orthogonal sequences to become the lamina and medulla neurons, respectively; the retinotopy of the lamina and medulla is established independently by these two cell populations, and generated by the chronotopic organization of the neuroblasts in each neuropil (Elofsson and Dahl, 1970; Meinertzhagen, 1973; Apitz and Salecker, 2015). As a consequence, the lamina and medulla have an inverted A-P axis and fibers connecting the two neuropils make the first optic chiasm with the axons crossing in the middle. Likewise, in OCH2, fibers connecting the medulla and lobula decussate and the A-P axes of the two neuropils are thereby inverted. Three separate cell populations of the IPC, along with the glial precursor cell (GPC) areas located at the dorsal and ventral tips of the OPC, generate neurons with cell bodies in the proximal medulla and the lobula complex (Apitz and Salecker, 2014, 2015; Suzuki et al., 2016a, b). Although cell types derived from these precursors are not completely known, it is reasonable to assume that the retinotopy of the lobula is determined independently from the medulla’s retinotopy before the Tm cells project from the medulla. If this proves to be true, the growth of the neurites between the lobula and lobula plate (as sheets) would seem to precede the neuronal projections from the medulla to the lobula complex (as bundles). Even though some neurons of the same type (e.g., T2 and T3 cells) have been shown to grow simultaneously, forming bundles in OCH2 during development (Ngo et al., 2017), the growth order of medulla-lobula neurons and medulla-lobula plate neurons among the bundles of neurites cannot be determined from the projection patterns that are their result. The growth order will require independent developmental studies for validation.

The four nested neuropils in the OL are a widely shared characteristic among insect and malacostracan crustacean species (Strausfeld, 2012). Comparative neuroanatomy and evolutionary studies suggest that the ancestral species of this group could have had a simpler configuration of their optic lobes, each with only two neuropils connected with uncrossed fibers, as seen in current non-pancrustacean arthropods including Scutigeromorpha (Elofsson and Dahl, 1970; Sinakevitch et al., 2003; Strausfeld, 2005, 2012).

A part of the neurons that form the OCH2 derive from the IPC. The IPC neuroepithelium is subdivided into two domains; proximal and surface IPCs. The proximal IPC neuroepithelium produces streams of migratory progenitors, which eventually turn into the third region, the distal IPC. These three subdomains are already visible in third-instar larvae (Apitz and Salecker, 2015). Among them, the surface IPC is known to develop into lobula neurons, while neuroblasts of the distal IPC initially generate the distal cells (C2, C3, T2, T2a, and T3 cells), and later switch to produce the T4 and T5 cells (Apitz and Salecker, 2015, 2018). Lineages and patterns of differentiation of other lobula plate neurons, including Y cells, LPLC cells, and lobula plate intrinsic cells, remain largely unknown. The lobula plate is connected to the medulla with uncrossed fibers, while fibers connecting the medulla and lobula decussate in OCH2 so as to invert the A-P axis.

Motion detection would have become especially important with the acquisition of flight among pterygote insects. They require motion detection in the vertical axis, and two of the T4 and T5 subtypes, c and d, mediate information that signals vertical motion (Joesch et al., 2010; Maisak et al., 2013). In the course of development, the expression of *dachshund* (*dac*) and *atonal* is required for differentiation of T4 and T5 cells (Apitz and Salecker, 2018). Dac is later downregulated in the subtypes c and d, while its expression is maintained in a and b. This fact indeed suggests that the subtypes a and b, which process horizontal movement information, could be the default fate of T4 and T5 (Apitz and Salecker, 2018), and the other two subtypes may have arisen later to complement spatial motion information processing. In *Neohelice granulata*, a benthic crab species, only two subtypes of the T4 and T5 cells, one each, are found. These are thought to process primarily horizontal movements as the animal moves over a flat surface, and they might be functionally correspond to the T4/T5 subtypes a and b in the fly (Bengochea et al., 2018). These observations on T4 and T5 cells imply that duplication of cell populations may have played a significant role in diversifying cell types in the optic lobe and in adapting to various visual environments.

### Architectural Advantage of the Chiasm for Visual Information Processing

**FIGURE 6:**
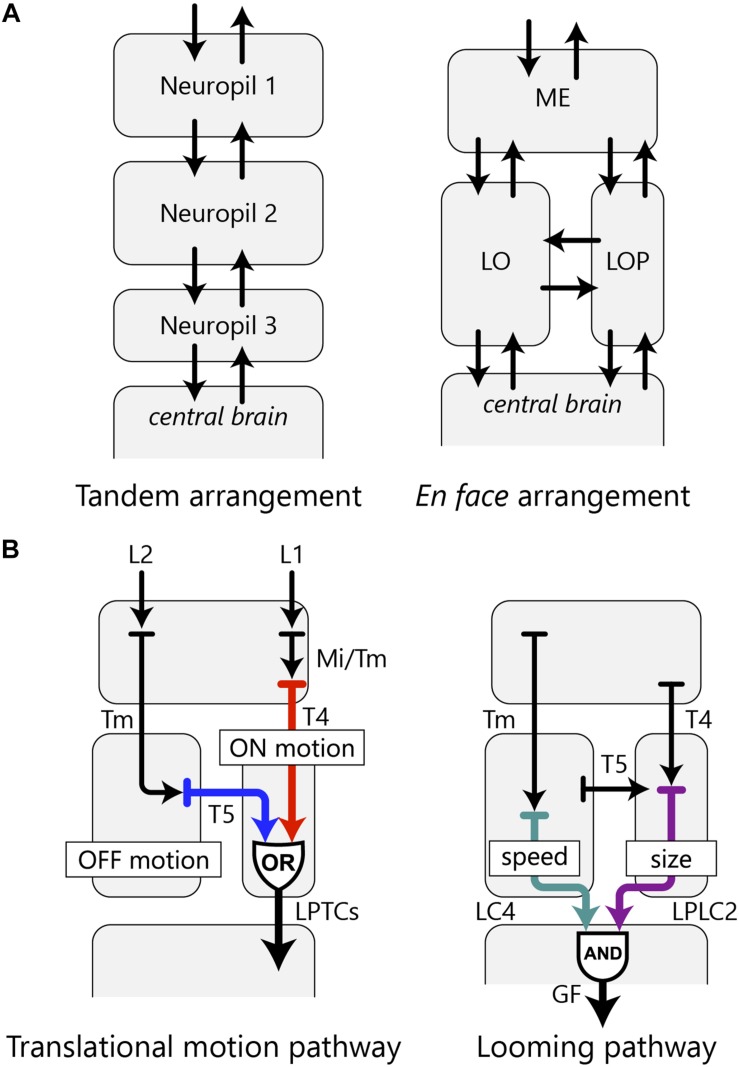
OCH2 and the arrangement of the optic lobe neuropils. **(A)** A hypothetical tandem arrangement (left) compared with the actual *en face* arrangement (right) of the optic lobe neuropils. The *en face* arrangement has more possible pathways between the neuropils as well as between the optic lobe and the central brain. **(B)** Two examples of information integration in the fly’s visual system accomplished by parallel information processing in multiple neuropils. The translational motion-processing pathway implements an OR operation, whereas the looming pathway implements an AND operation with the optic lobe neurons.

When the three retinotopic neuropils are arranged *en face*, one of the pathways between the neuropils necessarily crosses to form a decussation. The *en face* arrangement has the advantage in wiring economy to a simpler alternative tandem layout of the neuropils, because the number of possible connections between neighboring neuropils and the central brain is larger in the *en face* arrangement (Figure 6A). In the fly, as well as in many other insects and malacostracans, the lobula and lobula plate lie face to face, arranged in parallel as downstream neuropils to the medulla, rather than three consecutive neuropils connected in tandem. Some insect clades, such as ancient Odonata and Archaeognatha (jumping bristletails), have a tandem-like arrangement of the optic lobe neuropils, but these species have a chiasm with a fiber crossing between the medulla and lobula, and parallel fibers connecting the medulla and lobula plate (Strausfeld, 2012). Other groups, such as Hymenopterans, do not have an independent lobula plate and instead this region is functionally fused to the lobula. This lobula-lobula plate neuropil can be anatomically separated into two subsets of layers which correspond to the two individual neuropils, and that arrangement also maintains parallel and crossing connections with the medulla (Cajal and Sánchez, 1915; Strausfeld, 2012). So the optic lobe neuropils of these species seem homologous with those of other insects and probably with those of some crustacean groups as well. All do, however, require far more careful analysis of their cell types than is currently available, and that would thereby support safer, more detailed comparisons with Diptera.

Connecting the three neuropils via fibers of the OCH2 enables more complex visual information processing in these neuropils with the shortest path length. For example, ON-edge and OFF-edge motion information is detected independently by the T4 and T5 cell arbors in the proximal medulla and lobula, respectively, and eventually integrated at the lobula plate, with both cells sending output to the lobula plate tangential cells (LPTCs) (Joesch et al., 2010; Mauss et al., 2015; Takemura et al., 2017; Shinomiya et al., 2019). This entire motion pathway therefore comprises a circuit that implements an OR operation with independent sources of motion information from two neuropils, as its inputs (Figure 6B). In another case, two types of VPNs, the lobula columnar 4 (LC4) and lobula plate-lobula columnar 2 (LPLC2) cells, extract distinct visual features in the lobula and lobula plate, respectively, and both provide inputs to the giant fiber neuron in the ventrolateral protocerebrum, evoking the escape response (Otsuna and Ito, 2006; Klapoetke et al., 2017; Ache et al., 2019). This pathway constitutes an AND circuit implemented by VPNs of the lobula complex, with LC4 encoding object speed and LPLC2 encoding object size; both neurons provide inputs to the giant fiber neuron which sums signals from both pathways to drive escape response from a looming object (Figure 6B; Ache et al., 2019).

Insect and malacostracan species generally have larger bodies compared with those in other Pancrustacean arthropod clades, such as Branchiopoda, Copepoda, and Ostracoda, and they generally perform quick, active movements during food intake and escape behavior. We suggest that the highly sophisticated visual system with four optic lobe neuropils has been selected for, and has enabled, the behavioral characteristics of these species, to which insects and malacostracans added active three-dimensional movements during the course of their evolution. Evolution of the visual system, especially the duplication of the optic lobe neuropils and the formation of the chiasmata enabled, we further suggest, an increase in body mass and a more varied range of visual and visuomotor behaviors.

## Data Availability Statement

The datasets generated for this study are available on request to the corresponding author.

## Author Contributions

KS and IM: conceptualization, writing – original draft, writing – review and editing, and funding acquisition. KS, JH, and SP: methodology. KS: formal analysis. KS, AN, and IM: investigation. KS, SM, and MW: data curation. SP and IM: supervision. JH, SP, and IM: project administration.

## Conflict of Interest

The authors declare that the research was conducted in the absence of any commercial or financial relationships that could be construed as a potential conflict of interest.

## Footnotes

1 https://github.com/janelia-flyem/NeuTu
